# Expression patterns of ciliopathy genes *ARL3* and *CEP120* reveal roles in multisystem development

**DOI:** 10.1186/s12861-020-00231-3

**Published:** 2020-12-09

**Authors:** L. Powell, M. Barroso-Gil, G. J. Clowry, L. A. Devlin, E. Molinari, S. A. Ramsbottom, C. G. Miles, J. A. Sayer

**Affiliations:** 1 Translational and Clinical Research Institute, Newcastle University, Central Parkway, Newcastle upon Tyne, NE1 3BZ UK; 2 Biosciences Institute, Newcastle University, Framlington Place, Newcastle upon Tyne, NE2 4HH UK; 3 The Newcastle Hospitals NHS Foundation Trust, Freeman Road, Newcastle upon Tyne, NE7 7DN UK; 4 National Institute for Health Research Newcastle Biomedical Research Centre, Newcastle upon Tyne, NE4 5PL UK

**Keywords:** CEP120, ARL3, Foetus, Development, Retina, Kidney, Brain, RNAscope

## Abstract

**Background:**

Joubert syndrome and related disorders (JSRD) and Jeune syndrome are multisystem ciliopathy disorders with overlapping phenotypes. There are a growing number of genetic causes for these rare syndromes, including the recently described genes *ARL3* and *CEP120*.

**Methods:**

We sought to explore the developmental expression patterns of *ARL3* and *CEP120* in humans to gain additional understanding of these genetic conditions. We used an RNA in situ detection technique called RNAscope to characterise *ARL3* and *CEP120* expression patterns in human embryos and foetuses in collaboration with the MRC-Wellcome Trust Human Developmental Biology Resource.

**Results:**

Both *ARL3* and *CEP120* are expressed in early human brain development, including the cerebellum and in the developing retina and kidney, consistent with the clinical phenotypes seen with pathogenic variants in these genes.

**Conclusions:**

This study provides insights into the potential pathogenesis of JSRD by uncovering the spatial expression of two JSRD-causative genes during normal human development.

**Supplementary Information:**

The online version contains supplementary material available at 10.1186/s12861-020-00231-3.

## Background

Joubert syndrome and related disorders (JSRD) are a group of autosomal inherited ciliopathies that are characterised as a cerebello-retinal-renal phenotype, and have an incidence rate of 1:80,000–100,000 live births [1–3]. The hallmark brain phenotype is a “molar tooth sign” shown on axial brain MRI, caused by cerebellar vermis hypoplasia and other mid and hindbrain malformations [4]. These defects often cause symptoms of hypotonia, ataxia and intellectual disability in patients [5]. The retinal and renal phenotypes associated with JSRD have a lower incidence rate and vary in severity. Renal disorders occur in ~ 25% of patients, often presenting as corticomedullary cysts, interstitial fibrosis, or tubulointerstitial kidney disease [5]. The renal component is progressive and can lead to end-stage renal disease [6]. Ocular phenotypes of retinitis dystrophy, retinitis pigmentosa, oculomotor apraxia, and ptosis are common in patients, and as with the renal aspects of JSRD are often progressive in nature [7].

Currently, there are more than 35 genes that are known to cause JSRD (https://www.omim.org/phenotypicSeries/PS213300). The syndrome is caused by defects of the primary cilia, which are found on most mammalian cells [8]. Primary cilia act as a cellular antenna to transduce extracellular signals such as mechanical flow, chemical stimulation, and key signalling pathways (including Hedgehog, Wnt, and PDGF) into the cell [9–13]. Due to the multi-organ involvement, varying phenotypes, and multitude of genes known to cause JSRD there is great heterogeneity within the syndrome and overlap with closely related ciliopathies including Bardet-Biedl syndrome (https://omim.org/phenotypicSeries/PS209900) and Jeune syndrome (https://omim.org/phenotypicSeries/PS208500) [14]. Recently discovered genetic causes of JSRD include *ARL3* [15] and *CEP120* [16, 17]; the fact that their encoded proteins have such divergent roles within the primary cilium demonstrates the complexity underlying this group of related disorders.

ADP-ribosylation factor-like 3 (ARL3), a RAS superfamily member, is a low molecular weight GTP-binding protein [18] that cycles between inactive GDP-bound and active GTP-bound states to release cargo from their carriers in the cilium [19]. ARL3 interacts with its Guanine Exchange Factor ARL13B in the cilium [20] and GTPase Activating Protein RP2 at the basal body of the cell [21, 22]. *Arl3* knockout studies in mice demonstrate a multi-organ ciliopathy phenotype, including kidney cysts, liver fibrosis and retinal disease with photoreceptor cell degeneration [23–26]. Recently, two families affected by JSRD have been identified, presenting with ciliopathy phenotypes [15]. The underlying genetic cause was shown to be missense mutations in *ARL3*, which affect an amino acid residue involved in the interaction between ARL3 and ARL13B [15].

Centrosomal protein of 120 kDa (CEP120) is a centrosomal protein involved in centriole biogenesis, including centriole duplication, assembly [27, 28], elongation [29–31] and maturation [28]. CEP120 also interacts with other centrosomal proteins including CPAP [29, 30], SPICE1 [29], Talpid3 [28, 31] and C2CD3 [31]. CEP120 was found to be expressed ubiquitously in murine embryonic tissues such as the brain, kidney and lungs. Additionally, Cep120 was observed to be highly expressed in embryonic mouse brain compared to postnatal or adult mouse brain [32]. Inactivation of *CEP120* in the mouse central nervous system results in hydrocephalous and cerebellar hypoplasia [28]. *CEP120* mutations have been shown to cause JSRD and Jeune syndromes [16, 17], and overlapping ciliopathy phenotypes such as tectocerebellar dysraphia with occipital encephalocele (TCDOE), Meckel syndrome (MKS) and oro-facial-digital (OFD) syndromes [17].

The developmental expression patterns of *ARL3* and *CEP120* in humans is not known. In order to explore this, we used an RNA in situ detection technique called RNAscope to compare and contrast the developmental spatial expression of these new and divergent causes of JSRD. We successfully characterised *ARL3* and *CEP120* expression patterns in human embryos and foetuses in collaboration with the MRC-Wellcome Trust Human Developmental Biology Resource (HDBR). This study provides insights into the potential pathogenesis of JSRD by uncovering the expression pattern of two JSRD-causative genes during normal human development.

## Methods

### RNAscope studies

Characterisation of *ARL3* and *CEP120* expression patterns was performed in human embryonic tissue using samples obtained from the MRC-Wellcome HDBR. Formalin fixed paraffin embedded sections of human embryonic and foetal tissue were prepared using 10% neutral buffered formalin and fixed for 32 h at room temperature. Samples were then prepared for the RNAscope assay, a RNA in situ detection platform for detection of target RNA within intact cells, as per manufacturers’ instructions [33, 34]. An RNAscope 2.5 Assay RED was employed with 20 paired probes across nucleotides 169–1570 (NM_004311.3) and 115–1133 (NM_001166226.1) for detection of *ARL3* and *CEP120*, respectively and counterstained with Methyl Green.

Whole human embryo sections of 8 post-conception weeks (PCW), (equivalent to Carnegie Stage 23) were analysed, along with hindbrain (14PCW and 19PCW), eye (14PCW), kidney and adrenal gland (14PCW and 18PCW). A negative RNAscope 2.5 HD Assay Red control (*dapB*, a bacterial gene which is not expressed in human tissues) was performed (Supplementary Figure 1). In addition, the RNAscope 2.5 HD Assay RED was performed with a positive control (*KI67*, a cell proliferation marker). KI67 is a nuclear protein commonly used as a proliferation marker, which is expressed in cycling cells and is associated with cellular proliferation. It is encoded by the gene marker of proliferation Ki-67, *MKI67. ARL3* and *CEP120* human expression patterns were analysed using the HDBR image server (Leica Biosystems).

### Clinical phenotypes and sequence analysis

Reported clinical phenotypes associated with *ARL3* and *CEP120* mutations were reviewed within OMIM (https://omim.org/). Putative Arl3 and Cep120 orthologues were identified using BLASTP, with human ARL3 (isoform a, NP_004302.1) and CEP120 (NP_694955.2) transcripts as the query sequences within NCBI (https://www.ncbi.nlm.nih.gov/). Additional databases including Flybase https://flybase.org/), Wormbase (https://www.wormbase.org/) and Phytozome (https://phytozome.jgi.doe.gov/) were also queried using BLAST.

## Results

### Clinical phenotypes of ARL3 and CEP120 patients

Biallelic mutations in both *ARL3* and *CEP120* mutations are rare causes of ciliopathy syndromes. A comparison of the known phenotypes associated with *ARL3* and *CEP120* mutations is shown in Table 1. This overview reveals that mutations in *CEP120* are at present associated with severe phenotypes including MKS but also that single heterozygous changes in *ARL3* are sufficient to cause retinal-limited phenotypes. *ARL3* is highly conserved, with homologs present in *C. elegans*, *C. reinhardtii* and *D. melanogaster* whereas *CEP120* appears not to have homologs within these lower organisms (Supplementary Table 1). Known protein localisation within the cell of both ARL3 within the ciliary axoneme, and CEP120 in the centrosomes, are consistent with their role in ciliopathy syndromes (Supplementary Table 2).

**Table 1:** Comparison of the known phenotypes associated with *ARL3* and *CEP120* mutations

	Patients with ***ARL3*** related ciliopathy	Patients with ***CEP120*** related ciliopathy
**Number of affected patients reported and presenting phenotypes**	4 patients reported with JSRD secondary to biallelic changes [15] 4 patients with retinitis pigmentosa secondary to monoallelic changes [35, 36]	4 patients with JSRD 4 patients with JATD 1 foetus with MKS 1 foetus with TCDOE [16, 17]
**Brain imaging findings**	Molar tooth sign	Molar tooth sign
**Intellect**	Developmental delay Psychomotor delay	Developmental delay Cognitive impairment
**Skeletal**	No known associated phenotypes	Severely narrow chest Skeletal dysplasia Small and horizontal ribs Short limbs Polydactyly Synpolydactyly
**Mobility**	Ataxic gait Hypotonia	Truncal ataxia Hypotonia
**Eye**	Night blindness Bilateral vision loss Retinal dystrophy Ocular motor apraxia	Microphthalmia Duane syndrome Strabismus
**Kidney**	Cystic dysplastic kidney Bilateral renal scarring Recurrent urinary tract infections	Cystic dysplastic kidney

*JSRD* Joubert syndrome and related disorders; *JATD* Jeune asphyxiating thoracic dystrophy; *MKS* Meckel syndrome; *TCDOE* tectocerebellar dysraphia with occipital encephalocele

### ARL3 and CEP120 are expressed in early human brain development

**Fig. 1:**
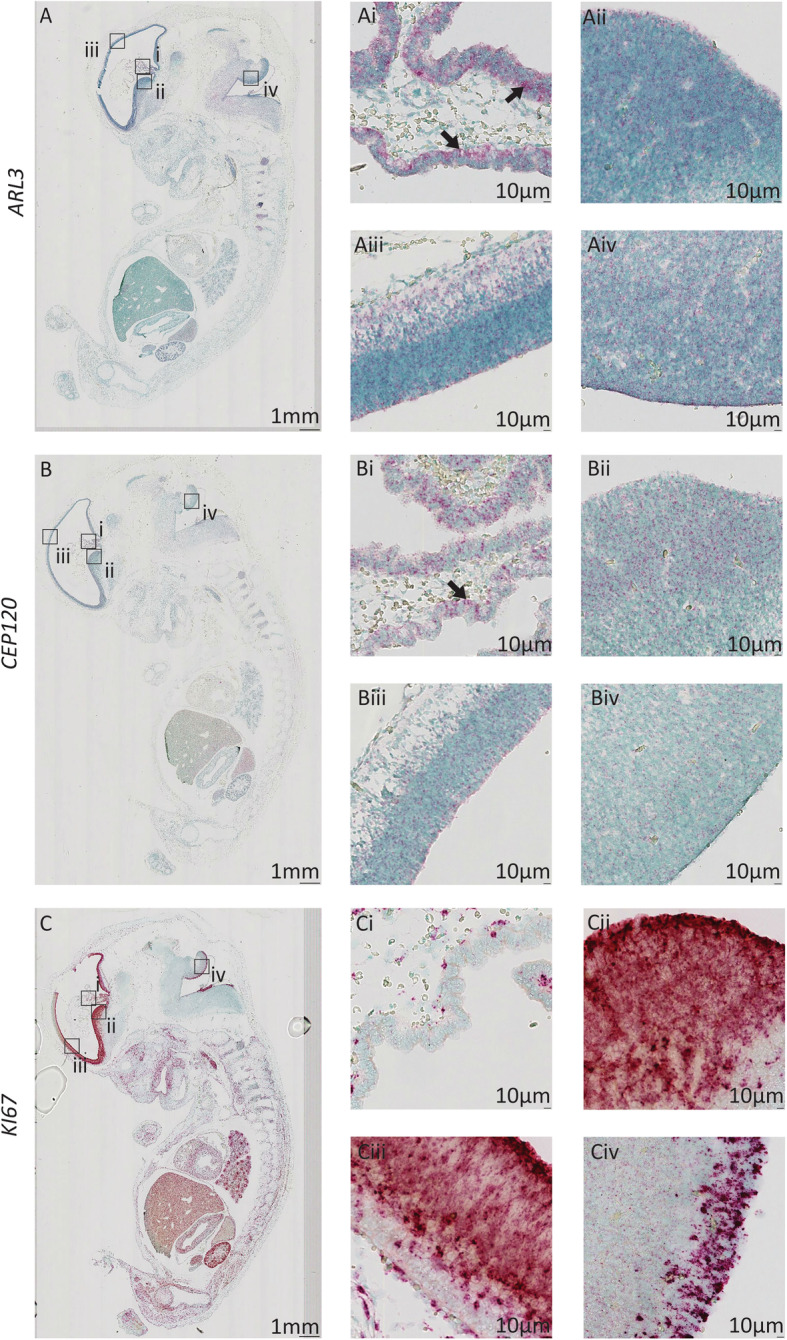
Expression pattern of *ARL3* and *CEP120* in the human brain during early development*.* Sagittal sections of 8PCW-stage human embryos stained using RNAscope to show expression of *ARL3* (**a**) (red), *CEP120* (**b**) (red) and *KI67* (**c**) (red), counterstained with Methyl Green. **Ai** and **Bi** *ARL3* and *CEP120* are expressed within cells of the choroid plexus (arrow). (Ci) *KI67* expression is minimal in the choroid plexus. **Aii**-**Aiv** and (**Bii**-**Biv**) Expression of *ARL3* and *CEP120* is seen in the ventricular radial glia progenitor cells including the ventricular zone of the ganglionic eminences (**Aii** and **Bii**), cerebral cortex (**Aiii** and **Biii**), and rhombic lip (**Aiv** and **Biv**). **Cii**-**Civ** Expression of *KI67* is seen in the ventricular zone of the ganglionic eminences, cerebral cortex, and hindbrain

In 8PCW human brain tissue, the expression of *ARL3* and *CEP120* is remarkably similar. There is expression of both genes in the choroid plexus (Fig. 1Ai and 1Bi), which appears to favour the luminal facing surface of the tissue, especially for *ARL3*. The cell proliferation marker *KI67* does not share this same expression pattern in the choroid plexus (Fig. 1 Ci). This specific expression pattern of *ARL3* and *CEP120* in luminal-facing cells is continued throughout the developing brain where both genes exhibit expression throughout the ventricular zone of the ganglionic eminences, cortical wall, and the hindbrain including the rhombic lip (Fig. 1Aii-Aiv and 1Bii-Biv). There is specific expression of *ARL3* and *CEP120* in the layer of cells forming the apical surface in each tissue, facing into the ventricular space. Expression of *KI67* is seen throughout these tissues (Fig. 1Cii-Civ), with specific expression in the apical layer consistent with this being the major site of cell division (cells in G2/M1 phase of cell cycle) in the ventricular zone [37].

### Expression of ARL3 and CEP120 is maintained in the developing cerebellum

**Fig. 2:**
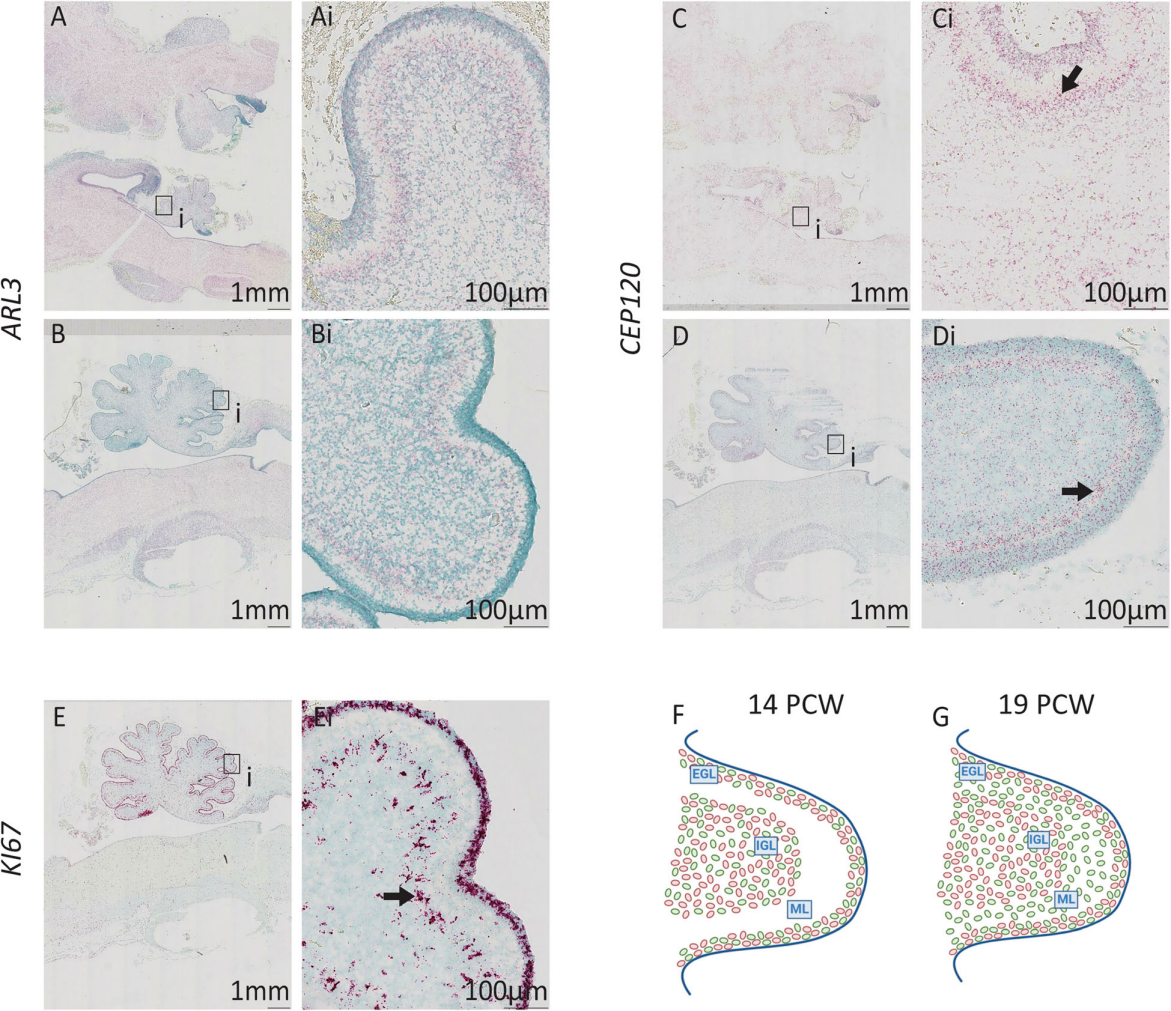
Expression of *ARL3* and *CEP120* in the developing human hindbrain. Sagittal sections of 14PCW (**a** and **c**) and 19PCW (**b**, **d** and **e**) human brain stained using RNAscope to show *ARL3* expression (**a** and **b**) (red), *CEP120* (**c** and **d**) (red) and *KI67* (**e**) (red), countered stained with Methyl Green. **Ai** and **Ci** Expression of *ARL3* and *CEP120* is evident in the cerebellum at 14PCW. **b** and **d** *ARL3* and *CEP120* expression in the cerebellum at 19PCW. **Bi** Cerebellar expression of *ARL3*. **Di** Cerebellar molecular layer (arrow) expression of *CEP120*. **e** and **Ei** Hindbrain *KI67* expression at 18PCW shows tissue is proliferative. **f** and **g** Schematic diagrams of developing cerebellum at (**f**) 14PCW and (**g**) 19PCW. Expression of *ARL3* is shown in red and *CEP120* in green. EGL, external granule layer; IGL, internal granule layer; ML, molecular layer

In the human cerebellum at 14PCW there is expression of *ARL3* and *CEP120*. Both genes have strong expression in the external and internal granule cell layer (EGL and IGL) the developing cerebellum (Fig. 2Ai and Ci). Expression in the EGL and IGL is seen at 19PCW for *ARL3* (Fig 2Bi) however, *CEP120* expression is predominantly localised in the EGL and the molecular layer (ML) of the cerebellum at 19PCW (Fig. 2Di). Strong expression of *KI67* is seen throughout the EGL in particular, but also the IGL at 19PCW, indicating the tissue is proliferative (Fig. 2Ei). *ARL3* and *CEP120* are therefore widely expressed in the cerebellum during development, with specific expression of *CEP120* in the ML which is predominantly occupied by the dendritic trees of Purkinje cells and the interacting parallel fibres of granule cells. As dendrites and axons contain low levels of mRNA, it is likely that *CEP120* expression is predominantly located in the sparse population of interneurons found in the molecular layer [38] or in immature granule cells migrating from EGL to IGL [39] suggesting a role for *CEP120* in these cell types [38].

### Expression of ARL3 and CEP120 in the developing eye

**Fig. 3:**
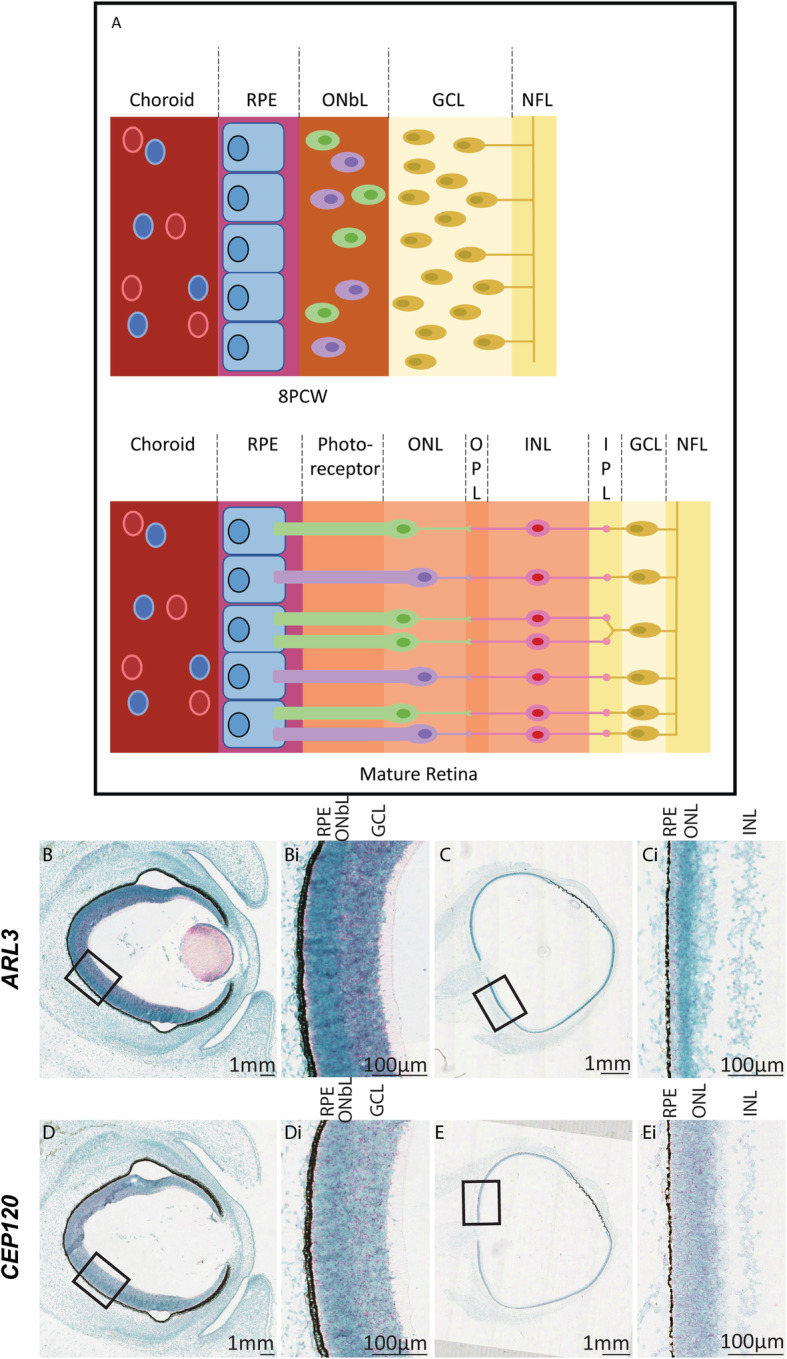
Expression of *ARL3* and *CEP120* in the developing human retina. **a** Schematic diagram of the development of the layers of the retina from 8PCW to the mature form (adapted from [40]). At 8PCW, not all of the layers are present in the retina. The ONbL is a transitionary layer containing retinal progenitor cells that will develop into various cell types such as photoreceptors, amacrine and bipolar cells; separating into the ONL, OPL, INL, and IPL (the IPL is sometimes visible at 8PCW). The GCL is thicker at 8PCW due to the migration of cells. The mature retina can be divided into layers. The RPE is at the very back of the eye and assists in the removal of waste products from the photoreceptor cells, which transduce light. The ONL, OPL, INL and IPL layers house intermediary cell bodies and dendrites that interact with ganglion cells in the GCL to convey the signal through the optic nerve, formed in the NFL, to the brain (reviewed in [41]). **b** Human sections of developing eye at 8PCW (**b** and **d**) and 14PCW (**c** and **e**) stained using RNA Scope to show ARL3 expression (**b** and **c**) (red) and CEP120 (**d** and **e**) (red) counterstained with Methyl Green (**Bi** and **Di**). There is a gradient of ARL3 (**Bi**) and CEP120 (**Di**) expression in the retina at 8PCW across multiple retinal layers including the ONbL. At 14 PCW, ARL3 (**Ci**) and CEP120 (**Ei**) expression is localised across multiple layers including the photoreceptor cell layer, just below the RPE layer (arrows). GCL, ganglion cell layer; INL, inner nuclear layer; IPL, inner plexiform layer; NFL, nerve fibre layer; ONL, outer nuclear layer; OPL, outer plexiform layer; ONbL, outer neuroblastic layer; RPE, retinal pigment epithelium

The human retina can be divided into nine layers based upon the cell types that occupy each layer (Fig. 3a), with the retinal pigment epithelial (RPE) and photoreceptor layers at the outermost part of the eye [39]. At 8PCW, the retinal layers are not well defined with only a ganglion cell layer separated from a layer of mostly immature neuroblasts with a few photoreceptor cells by a thin inner plexiform layer [40]. At this stage *ARL3* and *CEP120* show expression throughout the developing retina, with high expression within the retinal ganglion cells and the photoreceptor layer (Fig. 3Bi and Di). At 14PCW, the retinal layers are maturing [39] which is reflected in the expression pattern of both *ARL3* and *CEP120.* Clear expression of both genes is still seen in all layers of the retina, although to a lesser extent in the plexiform and nerve fibre layers due to reduced cell density in these areas (Fig. 3 Ci and Ei).

### Expression of ARL3 and CEP120 in the developing dorsal root ganglia

**Fig. 4:**
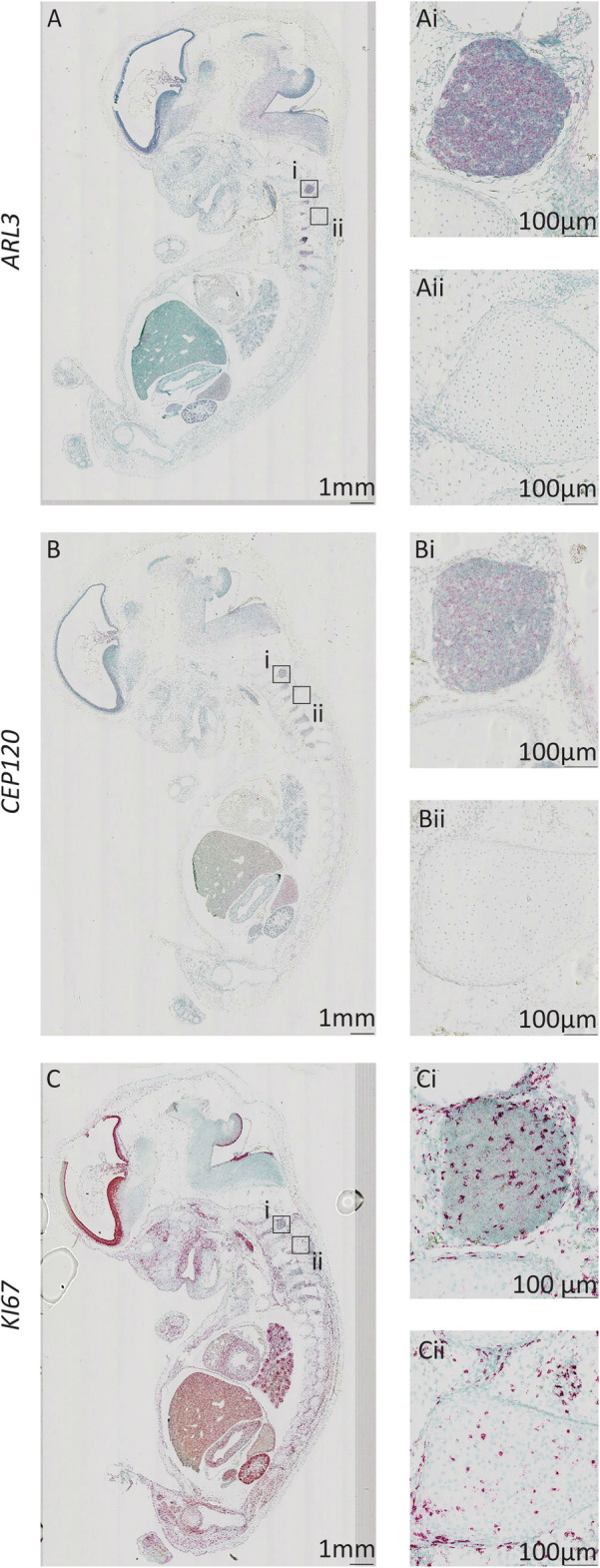
Expression of *ARL3* and *CEP120* in the developing human dorsal root ganglia Sagittal sections of 8PCW human embryos stained using RNAscope to show expression of *ARL3* (**a**) (red), *CEP120* (**b**) (red) and *KI67* (**c**) (red) counterstained with Methyl Green. *ARL3* and *CEP120* expression is shown within the dorasl root ganglia (**Ai** and **Bi** respectively), whereas surrounding tissue has low level expression of these genes (**Aii** and **Bii**). *KI67* expression is seen in the dorsal root ganglia (**Ci**) and surrounding tissues (**Cii**)

The dorsal root ganglia are formed by migrating neural crest cells and contain most of the body’s sensory neurones [42, 43]. Both *ARL3* and *CEP120* show expression in cells of the dorsal root ganglia, which are post-mitotic primary sensory neurons (Fig. 4 Ai-Aii and Bi-Bii). There is strong expression of *KI67* within limited number of cells in the dorsal root ganglia, presumably non-neuronal (Fig. 4 Ci-Cii).

### Expression of ARL3 and CEP120 in the developing kidney

**Fig. 5:**
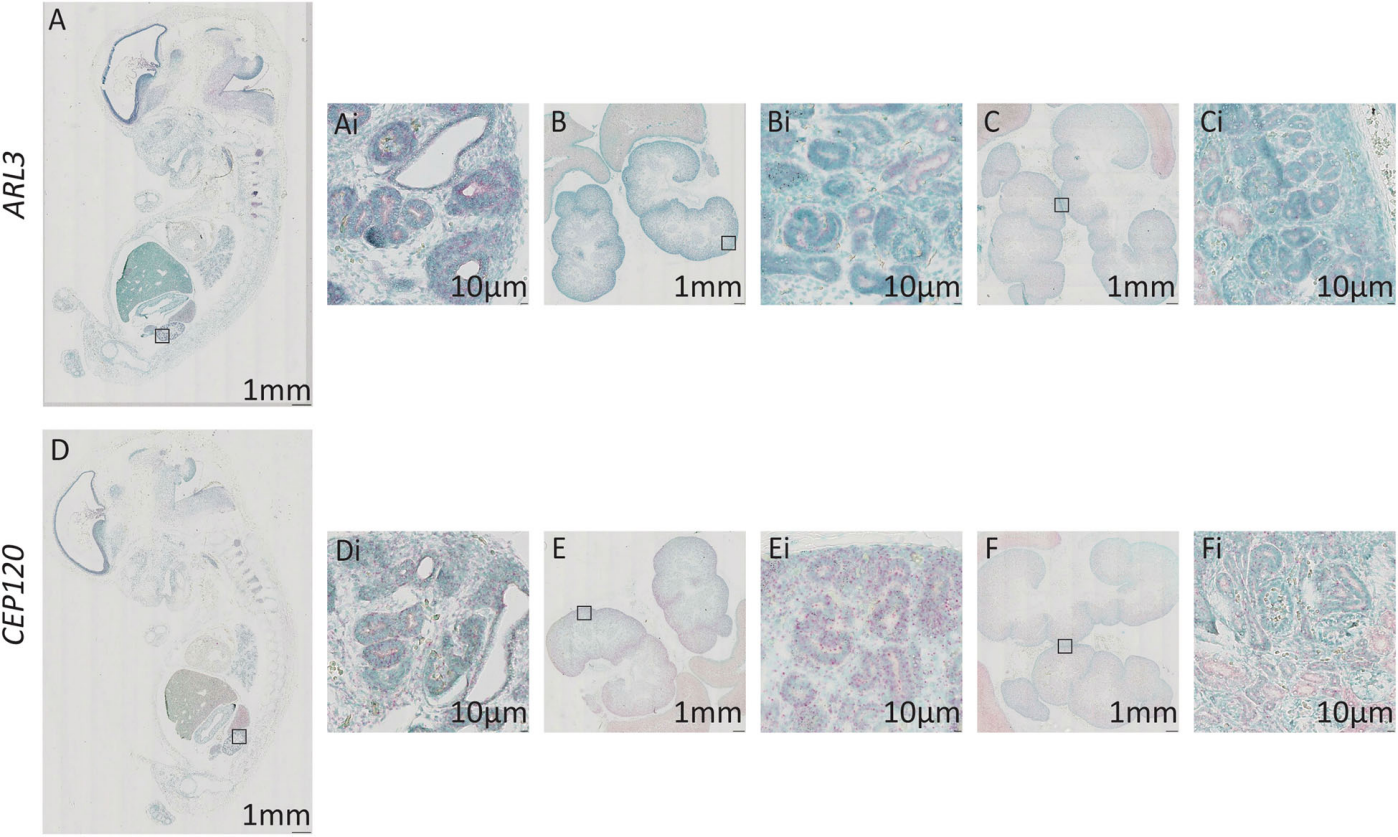
Expression of *ARL3* and *CEP120* in the developing human kidney. Sagittal sections of human kidney at 8PCW (**a** and **d**), 14PCW (**b** and **e**), and 18PCW (**c** and **f**) stained using RNAscope to show *ARL3* expression (**a**, **b**, **c**) (red) and *CEP120* (**d**, **e**, **f**) (red) and counterstained with Methyl Green. *ARL3* and *CEP120* expression at 8PCW (**Ai** and **Di**) is seen in the developing kidney cortex. *ARL3* and *CEP120* expression in the kidney cortex remain the same at 14PCW (**Bi** and **Ei** respectively). Ci and Fi shows persistent *ARL3* and reduced *CEP12*0 kidney cortex expression at 18PCW

In the developing human kidney at 8PCW, where there is strong renal cortical staining of *KI67* indicating cell proliferation (Supplementary Figure 2), there is expression of *ARL3* in cells within the developing cortical nephrons; this expression appears to be specifically oriented to the lumen of the structures (Fig. 5Ai). This expression pattern is maintained at 14PCW (Fig. 5Bi) and 18PCW (Fig. 5 Ci). Expression of *CEP120* is also seen in developing nephrons at 8PCW, however there is also expression in the renal cortex (Fig. 5Di). This expression pattern of *CEP120* is maintained at 14PCW (Fig. 5Ei) and 18PCW, although overall expression appears to have decreased at this time point (Fig. 5Fi).

### Expression of ARL3 and CEP120 in other major organs

In the developing human heart, lung and gut at 8PCW, there is very low levels of expression of *ARL3* and *CEP120* (Supplementary Figure 3). Expression of *ARL3* and *CEP120* is seen around the developing alveoli and at low levels in the developing bowel epithelia. The remaining organs of the developing embryo did not reveal prominent expression patterns.

## Discussion

Mutations in *ARL3* and *CEP120* are rare and relatively new causes of JSRD and other related ciliopathies. Human protein atlas data suggests that tissue expression of ARL3 protein is widely expressed, with highest expression scores seen in cerebellum and lowest in heart and skeletal muscle (https://www.proteinatlas.org/ENSG00000138175-ARL3/tissue). RNA expression is high in cerebral cortex, cerebellum, retina and kidney consistent with its known phenotypes. CEP120 protein expression is not annotated within the human protein atlas, whereas RNA is strongly expressed in the cerebellum (https://www.proteinatlas.org/ENSG00000168944-CEP120/tissue). We aimed to define expression of *ARL3* and *CEP120* during human development using the HDBR tissue bank employing a relatively new in situ hybridisation assay called RNAscope for the detection of target RNA within intact cells. We used *KI67* as a positive control although we recognise that expression of *KI67* is not homogeneous throughout each tissue. Our data provide an insight into the developmental expression of *ARL3* and *CEP120*. We show that both of these genes are expressed in key tissues (including retina, cerebellum and kidney) during development. This expression pattern fits with the multisystem disease phenotypes seen in patients with *ARL3* and *CEP120* mutations (Table 1). A similar approach, using the valuable HDBR tissue bank has been performed, using in situ hybridisation for studying the expression of *ARL13B* [44]*,* another cause of Joubert syndrome. Here *ARL13B* was detected at stage CS16 in the alar and basal plate of the myelencephalon, the mesencephalon and the metencephalon. At CS19 *ARL13B* was seen in the ventricular layer of the diencephalon and myelencephalon, the tegmentum of the pons and the cerebellar rhombic lips as well as the dorsal root ganglia. This pattern of expression is remarkably similar to the *CEP120* and *ARL3* data described here.

Expression of both *ARL3* and *CEP120* was minimal in developing cardiac, lung and gut tissues, consistent with lack of known phenotypes affecting these organ systems (Supplementary Figure 3). *ARL3* and *CEP120* encode proteins that are expressed in the primary cilia and basal body respectively (Supplementary Table 2) and pathogenic variants result in similar and overlapping phenotypes, including the cerebello-retinal-renal syndrome JSRD (Table 1). The number of patients with pathogenic variants in either *ARL3* or *CEP120* remains small, allowing a limited comparison of phenotypes, although skeletal manifestations (in particular short ribs/asphyxiating thoracic dystrophy phenotypes) seen in patients with *CEP120* mutations have not been documented in patients with *ARL3* mutations.

There were notable differences in evolutionary conservation between *ARL3* and *CEP120* (Supplementary Table 1). The ARL3 human protein shares greater than 90% identity with its two orthologous sequences (there is genomic duplication of *arl3*) in *Danio renio* (zebrafish), a well-studied model species in vertebrates. In contrast, CEP120 human protein only shares 57% identity with its single orthologous sequence found in zebrafish. Moreover, human ARL3 protein shares > 60% identity with its orthologues found in *Drosophila melanogaster*, *Caenorhabditis elegans* and *Chlamydomonas reinhardtii*. CEP120 is conserved in some vertebrate organisms but orthologues were not readily identified in invertebrates. There is a putative *CEP120* orthologue, UNI2, found in *Chlamydomonas reinhardtii*, but this has not as yet been confirmed as a functional ortholog [27, 44]. ARL3 is described in diverse eukaryotic organisms such as *Leishmania donovani* [45] and *Caenorhabditis elegans* [46] [47] where it has a functional role in the cilium/flagella. Despite these differences in evolutionary conservation, our results show that *ARL3* and *CEP120* have similar expression patterns during human development, specifically in the eye and dorsal root ganglia as well as during early brain development. Both genes are expressed throughout the retina during development, with expression in the RPE and photoreceptor layers, suggesting a role for both genes during retinal development. This is further supported by the numerous retinal phenotypes associated with mutations in *ARL3* [15–17]. Similarly, the specific expression of *ARL3* and *CEP120* in the dorsal root ganglia hints at a role for both genes in primary sensory neurone differentiation. A recurring pattern was the expression of both mRNAs on the luminal facing surface of the cerebral tissue (seen in the choroid plexus and ventricular zones of the cerebral cortex ganglionic eminences, and hindbrain) which could suggest a sensory role for the gene products of both the genes within the cilium of the ventricular lining of the brain.

Expression of *ARL3* and *CEP120* changes during development notably in the cerebellum and kidney. *ARL3* and *CEP120* are expressed throughout the cerebellum at 14PCW however, at 19PCW, ARL3 expression was predominantly in the IGL whereas *CEP120* was expressed in the EGL and ML of the cerebellum. This could imply that *ARL3* and *CEP120* are expressed in different cell populations of the cerebellum, *ARL3* in both immature and mature granule cells, and *CEP120* in immature, migratory granule cells and ML interneurones [48]. It has been previously reported in mouse studies that *Cep120* is required for proliferation of cerebellar neural progenitor cells [28] and is required for correct development of the embryo. Taken with these results, it suggests that *CEP120* expression is required for correct development of the cerebellum in humans.

Expression of *ARL3* and *CEP120* also differed in the developing kidney. The results showed that *ARL3* was specifically expressed in cells of the nephrons whereas *CEP120* was expressed in the nephrons as well as within cells in the developing renal cortex. This difference in expression could imply that *ARL3* has a more sensory/signalling function in luminal structures of the kidney, whereas *CEP120* has a more universal role in all cells as it is expressed more ubiquitously throughout the tissue.

The differences in gene expression may reflect the divergent functions of ARL3 and CEP120 proteins (Supplementary Table 2). As ARL3 is a trafficking protein involved in ciliary signalling [19, 49], it may only be expressed in actively signalling cells during certain points in development such as nephron progenitors and cells in the IGCL. In contrast, CEP120 is involved in building the centriole, and therefore cilium, [27, 29] and so will be expressed more widely within tissues, especially those with ciliated epithelia [50, 51].

In conclusion, we establish in human embryonic tissue expression patterns of *ARL3* and *CEP120* during development and provide insights into the wide phenotypic spectrum of mutations affecting *ARL3* and *CEP120* in humans. These studies will allow further investigations into tissue-specific mechanistic roles of *ARL3* and *CEP120* in human health and disease.

## Supplementary Information

**Additional file 1.**

## Data Availability

All data generated or analysed during this study are included in this published article and its supplementary information files. The datasets used and/or analysed during the current study are available from the corresponding author on reasonable request.

## Abbreviations

EGL: External granule cell layer
GCL: Ganglion cell layer
HDBR: Human Developmental Biology Resource
IGL: Internal granule cell layer
INL: Inner nuclear layer
IPL: Inner plexiform layer
JATD: Jeune asphyxiating thoracic dystrophy
JSRD: Joubert syndrome and related disorders
MKS: Meckel syndrome
ML: Molecular layer
NFL: Nerve fibre layer
OFD: Oro-facial-digital
ONbL: Outer neuroblastic layer
ONL: Outer nuclear layer
OPL: Outer plexiform layer
PCW: Post-conception weeks
RPE: Retinal pigment epithelium
TCDOE: Tectocerebellar dysraphia with occipital encephalocele
