# Supplementary material for: Expression patterns of ciliopathy genes ARL3 and CEP120 reveal roles in multisystem development
Source: BMC Dev Biol. 2020 Dec 9;20:26. doi: 10.1186/s12861-020-00231-3 (PMC7727171; doi:10.1186/s12861-020-00231-3)
Supplement: Supplementary file 1 — Additional file 1. [file 12861_2020_231_MOESM1_ESM.pdf]

Supplementary Materials

Expression patterns of ciliopathy genes *ARL3* and *CEP120* reveals roles in multisystem development

Powell L[1]*, Barroso-Gil M[1]*, Clowry GJ[2], Devlin LA[1], Molinari E[1], Ramsbottom SA[1], Miles CG[1] and Sayer JA[1,3,4]

	ARL3		CEP120		
Organism	ID	Identity %	ID	Identity %	Database
Homo sapiens	NP_004302.1	100	NP_694955.2	100	NCBI
Bos taurus	NP_001033656.1	98.35	NP_001071468.1	88.65	NCBI
Mus musculus	NP_062692.1	98.35	NP_848801.2	89.17	NCBI
Rattus norvegicus	NP_073191.1	97.25	NP_001178626.1	90.39	NCBI
Gallus	XP_421730.1	96.7	XP_015136305.1	72.03	NCBI
Xenopus tropicalis	XP_002938771.2	97.8	NP_001120380.1	65.74	NCBI
Erpetoichthys calabaricus	XP_028651567.1	95.05	XP_028661076.1	61.36	NCBI
Danio rerio	*NP_001038373.1	94.51	XP_017212877.1	57.67	NCBI
Gadus morhua	XP_030195485.1	94.51	XP_030226769.1	55.52	NCBI
Oryzias latipes	XP_011486618.1	93.96	XP_011477980.1	54.12	NCBI
Takifugu rubripes	XP_003978186.2	92.31	XP_011613677.2	52.08	NCBI
Callorhinchus milii	XP_007910451.1	97.8	XP_007897573.1	64.01	NCBI
Drosophila melanogaster	FBpp0083549	65.54	Not found		FlyBase
Caenorhabditis elegans	F19H8.3	63.39	Not found		WormBase (WS72)
Chlamydomonas reinhardtii	Cre04.g218250.t1.2	63.84	Not found		Phytozome

	Mammals
	Tetrapods (non-mammals)
	Euteleostomi (non-tetrapods)
	Vertebrate (non-euteleostomi)
	Bilateria (non-vertebrate)
	Eukaryota (non-bilateria)

Supplemental Table 1. Comparison of the homology of ARL3 and CEP120 amino acid sequences

*arl3b. Due to whole genome duplication events in teleost fish there are 2 transcripts of arl3 named arl3a NP_001315350.1 (with 95.05% identity with human ARL3) and arl3b NP_001038373.1 (with 94.51% identity with human ARL3).

	ARL3	CEP120
**Ciliary / Basal body Localisation**	Ciliary Axoneme (ARL3-GTP) (24)	Centrosome (asymmetrically localized to the daughter centriole) (27)
**Protein Domains**	GTPase (18)	3 consecutive C2 domains (C2A, C2B and C2C) and a coil-coiled domain (27, 44)
**Function**	Releases prenylated, farnesylated, geranylgeranylated and myristolyated cargo from their carriers (e.g. PDE6δ and UNC119a/b) in the cilium (19, 45, 51) (52)	Centriole duplication, assembly, elongation and maturation (27, 29)
**Interactors**	ARL13B (GEF) (20)<br>RP2 (GAP) (21)	TACCs (32), CPAP (29, 30), SPICE1 (29), Talpid3 (28), C2CD3 (31)

Supplemental Table 2. Comparison of the ciliary localisation, protein domains, function and protein interactors of the ARL3 and CEP120 proteins

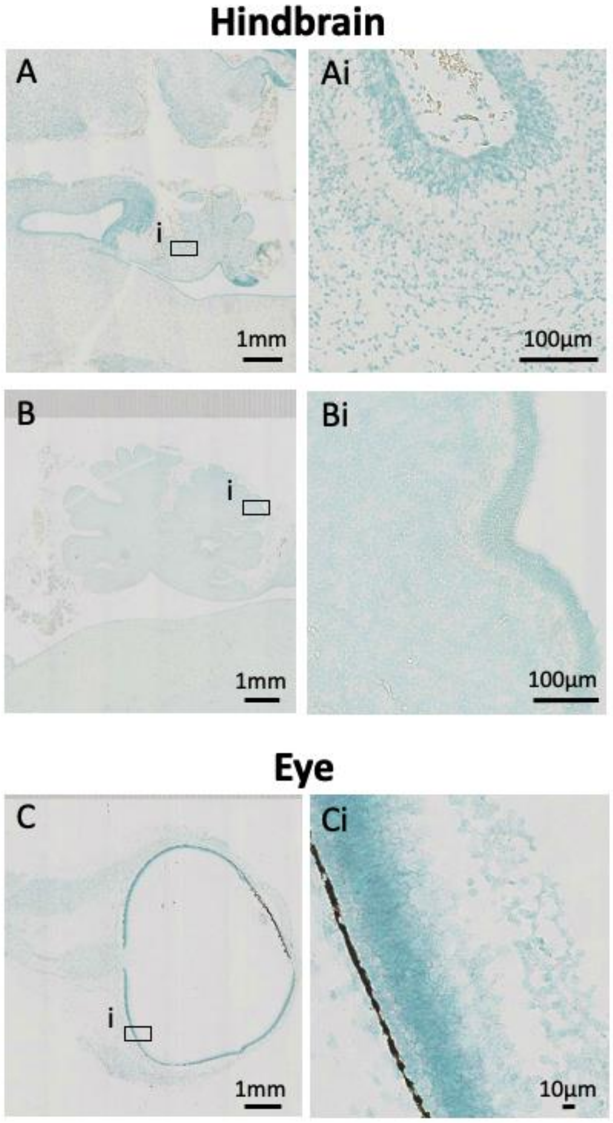

Supplementary Figure 1. Expression of *dapB* in the developing human hindbrain and eye

Sagittal sections of 14PCW (A) and 19PCW (B) human hindbrain and 14PCW (C) human eye stained using RNAscope to show *dapB* expression (red), counter-stained with Methyl Green. *dapB*, a bacterial gene, expression is used as a negative control. Expression of *dapB* is absent in the cerebellum at 14PCW (Ai) and 19 PCW (Bi) and the retina at 14PCW (Ci).

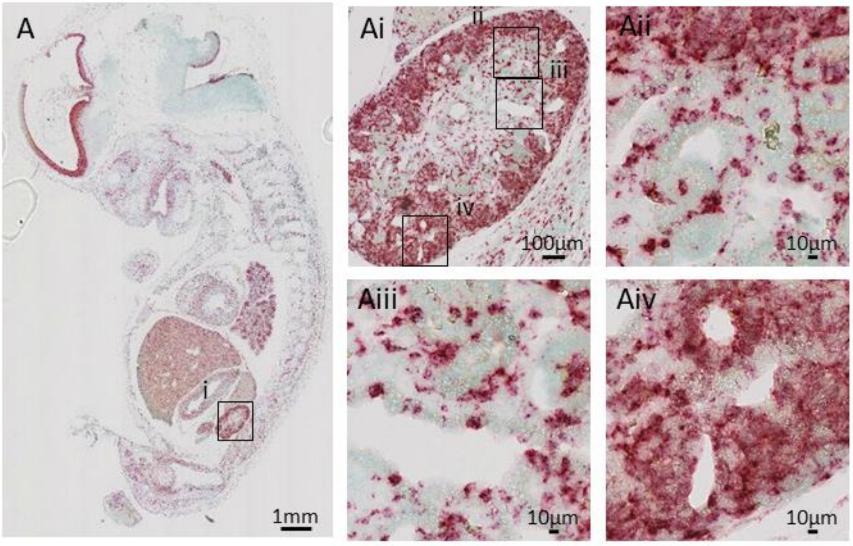

Supplementary Figure 2. Expression of *KI67* in the developing human kidney

Sagittal section of a kidney of a human embryo at developmental stage 8PCW, (A, Ai-Aiv) stained using RNAscope to show expression of *KI67* (red) counterstained with Methyl green.

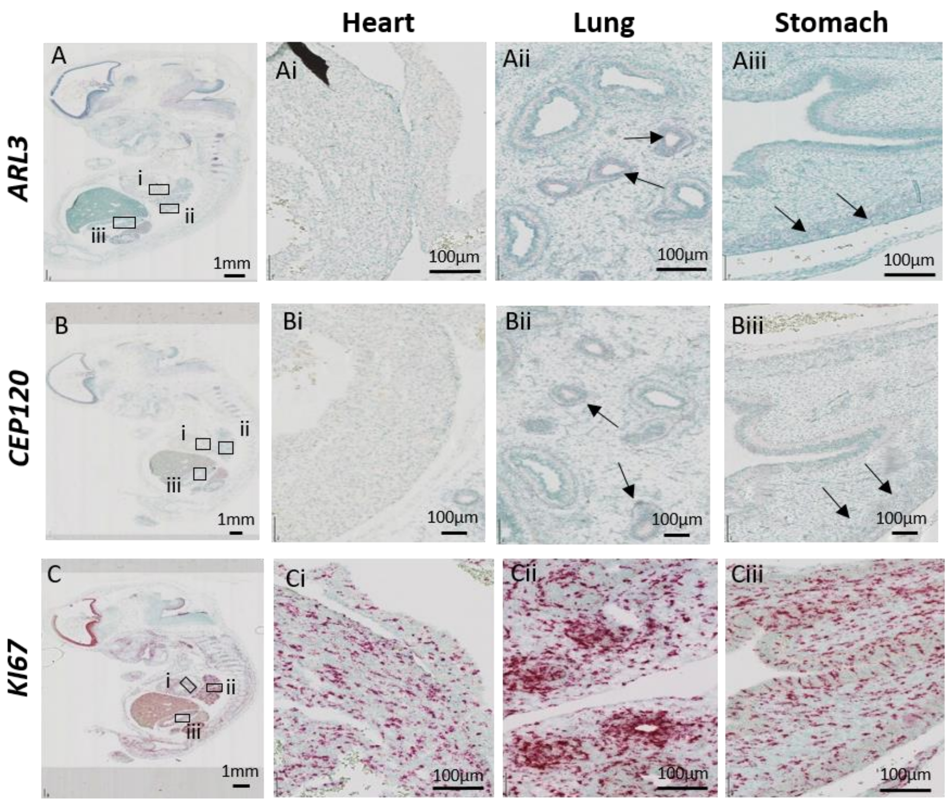

Supplementary Figure 3. *ARL3* and *CEP120* are not highly expressed in all developing tissues

Sagittal sections of human heart, lung and stomach at 8PCW stained using RNAscope to show expression of *ARL3* (A) (red), *CEP120* (B) (red) and *KI67* (C) (red) counterstained with Methyl Green. In the heart, neither *ARL3* (Ai) or *CEP120* (Bi) are highly expressed, whilst the proliferation marker *KI67* is expressed (Ci). There is some expression of *ARL3* and *CEP120* in the lung (Aii and Bii) around the alveoli (arrows), but not in the surrounding tissue. *KI67* is expressed throughout the lung (Cii). In the gut, *ARL3* (Aiii) and *CEP120* (Biii) are expressed weakly (arrows). *KI67* is expressed strongly throughout the developing gut (Ciii).
